# The Book World of Medicine and Science

**Published:** 1898-07-23

**Authors:** 


### The Book World of Medicine and Science.

A Century of Vaccination and What it Teaches. By W. Scott Tebb, M.A., M.D.Cantab., D.P.H., Surgeon to the Boscombe Hospital. Small octavo, 448 pages. Price 6s. (Swan Sonnenschein and Co., Limited. 1898.)

Like his father, Dr. W. Scott Tebb is an anti-vaccinationist, and in this he resembles Dr. W. J. Collins, whose father was a well-known opponent of vaccination a generation ago. Here the resemblance between the two men almost ceases, for Dr. Tebb exhibits none of the dialectic skill with which Dr. Collins endeavoured, in his dissent from the report of the Royal Commission, to make the worst appear the better case. Nor do we think that Dr. Collins, for want of better argument, would have demeaned himself by accusing his own profession of having defended vaccination for monetary considerations. That the charge is made in general terms, without either name or case in support of it, renders it all the more offensive. As to vaccination, Dr. Tebb says that "its risks, so long denied, are now on all hands admitted." The statement is disingenuous, or else the author has failed to convey his meaning clearly. "Its risks," as described by Dr. Tebb, will be "admitted" by hardly any medical men, and least of all by men like Drs. Acland and Coupland and Barlow, who have specially devoted themselves to inquiry into allegations of evil results. Dr. Tebb says that "the investigations of Creighton and Professor Crookshank have proved conclusively that cowpox is a disease radically different from that against which it is said to protect." But that is mere assertion on Dr. Tebb's part. The Royal Commission arrived at no such conclusion, and the conclusions of Dr. Creighton and Professor Crookshank themselves are greatly at variance with each other. Dr. Tebb, therefore, has no right to say that "many of my brethren" are "willing to acknowledge that there is no true pathological relation between cowpox and smallpox." The introduction of the word "acknowledge" here is an abuse of the English language. As regards variolous attack of unvaccinated persons in local epidemics, he says that this would strongly support a belief in vaccination "but for the fact that in localities where the vaccination law is vigorously carried out, the unvaccinated, as a class, will be found to consist largely of the outcasts of society —nomads whom the law has failed to reach—and of weakly children who, on account of their health, have been excused the operation. This class, therefore, is likely to furnish a disproportionate number of the victims of the epidemic." This argument will not bear analysis even "where the vaccination law is vigorously carried out," but why does Dr. Tebb not face the corresponding facts regarding towns in which vaccination is in abeyance? The spread of anti-vaccination is rapidly proving the fallacy of some of its own principal allegations. In Dewsbury and Leicester and Gloucester, the unvaccinated are not distinguished from the vaccinated by being outcasts and nomads and so forth, whilst as to weakly children, by all the theories of anti-vaccination the vaccinated in such towns ought to be the weaker, with their blood poisoned by so-called vaccinal "filth." Yet in these anti-vaccinating towns when small-pox invades a household it picks out the unvaccinated and passes by the vaccinated, to an extent which is only to be explained on the theory that vaccination makes the difference. What, then is the use of Dr. Tebb discussing the question on the basis of "localities where the vaccination law is vigorously carried out" unless because he can there let his imagination run riot as to nomads and outcasts and weakly children?

Regarding small-pox inoculation, we are told that "Dimsdale preferred inoculating from mild cases, and from arm to arm, for he says: 'If neither an inoculated patient is at hand, nor anyone in the neighbourhood has a distinct kind of the natural disease, a thread may be used in the common manner, provided the thread be very recently infected.'" But there is not a syllable here about arm to arm inoculation, and the passage only serves to show how readily Dr. Tebb arrives at conclusions on insufficient evidence. It happens that Dimsdale has left us in no doubt as to his practice, for he says that he "gives a preference to the matter taken during the eruptive fevers," and that he believes in "recent fluid matter." No recent fluid matter would be got from the inoculation pustule on the arm during the eruptive fever, and when Dimsdale did the most important inoculation of his life—that of the Empress of Russia—he used matter taken during the eruptive stage. Dr. Tebb also deals with Thornton's Stroud cases, inoculated with the Stonehouse matter, which were brought before the Royal Commission by Professor Crookshank, whose omission of the crucial words from a long quotation is only less extraordinary than his omission of a large part of a passage quoted from Sir James Y. Simpson's writings. Dr. Tebb gives the words which Professor Crookshank left out, but he gives no hint as to how essentially they affect his argument. Then he proceeds: "At the end of 1798, six months after the publication of Jenner's 'Inquiry' the case for vaccination stood thus: Most of the children's arms had ulcerated, and the variolous test, in the few cases in which it had been applied, had produced equivocal results." The line followed here, and even the words, seem to be suggested by Section 45 of the Statement of Dissent by Dr. Collins, beginning "Thus the matter stood," both passages, as it happens, coming immediately after reference to the Stonehouse lymph. Like Dr. Collins and Mr. Picton, Dr. Tebb here omits all reference to Pearson's "Inquiry Concerning the History of Cow-pox," published before the end of 1798, and furnishing most important evidence of the power of cow-pox in preventing small-pox. To say that "the case for vaccination stood thus" at the end of 1798 and to fail to call attention to Pearson's monograph is utterly unjustifiable from a historical point of view. The above remarks apply wholly to a few of the statements contained within the first 16 of the 448 pages of Dr. Tebb's book, and it seems quite unnecessary to follow him further through his "Century of Vaccination."

How to Select a Life Office. By G. M. Dent, F.S.S. Third Edition. (Manchester and London: John Heywood. Pp. 36. Price 1s.)

Mr. Dent's pamphlet in dialogue form is now well known. It is a frank and open advertisement of a large amount of research into the comparative value of life offices, and of the author's readiness to place his services as agent at his readers' disposal. His tables amply illustrate the relative security of seventy-five offices, and the return which the assurer may expect to get for his premium in each case. The different factors on which these points depend are clearly and judiciously stated, and the manner in which various offices cater, more particularly for assurers at different times of life, is also well set forth. The reader cannot fail to be convinced that there is no single office which is best for all classes of insurance, and he is bound to recognise that each individual case require careful consideration on its merits. As a means of warning assurers to look before they leap into the clutches of the insurance agent this little pamphlet is good.

